# Delayed Sudden Radial Artery Rupture After Left Transradial Coronary Catheterization: A Case Report

**DOI:** 10.1097/MD.0000000000000634

**Published:** 2015-03-13

**Authors:** Ciro Indolfi, Francesco Passafaro, Annalisa Mongiardo, Carmen Spaccarotella, Daniele Torella, Sabato Sorrentino, Alberto Polimeni, Vittorio Emanuele, Antonio Curcio, Salvatore De Rosa

**Affiliations:** From the Division of Cardiology (CI, FP, AM, CS, DT, SS, AP, VE, AC, SDR), Department of Medical and Surgical Sciences, Magna Graecia University; and URT-CNR (CI), Department of Medicine, Consiglio Nazionale delle Ricerche, Catanzaro, Italy.

## Abstract

Local complications at the radial access site are not frequent, hence its large diffusion as the preferred access route for endovascular procedures. However, in a time of fast widespreading, better comprehension of all potential complications becomes critical to facilitate their early recognition and the most appropriate treatment.

In this case report, we present for the first time a case of sudden massive bleeding at the left wrist, due to spontaneous gross rupture of the left radial artery bleeding 15 days after an endovascular procedure through a left radial arterial access. The patient had been readmitted to the hospital after evidence of local infection at the left wrist with loss of substance. The radial artery was patent with no evidence of pseudoaneurysm.

After sudden radial artery rupture, with massive bleeding and suspicion that the local infection could have reached the arterial wall, surgical hemostasis with artery ligation was obtained. Healing of the large wound was then efficiently speeded up using a negative pressure wound therapy.

This is the first case of macroscopic radial artery rupture associated with local wrist infection after arterial catheterization. After prompt surgical hemostasis, negative pressure wound therapy was very helpful in favoring healing of the large and deep wound.

## INTRODUCTION

The transradial approach (TRA) is becoming the preferred access site for coronary angiography, mainly for its safety profile.^1^ In fact, TRA is associated with a substantial reduction of access site complications as compared with the transfemoral access, along with an increased comfort to the patient and a shorter time to ambulation.^1^ These advantages are even larger during acute myocardial infarction, in which use of the TRA leads to a decrease in death.^1^

Although use of the TRA has been shown to be quite safe, even during the early phase of adoption,^2^ attention should be paid to the early identification and treatment of local complications—especially in a phase of fast widespreading—to prevent serious consequences. We describe the first case of local loss of substance associated with local infection at the radial access site with subsequent sudden massive bleeding 15 days after arterial cannulation, due to rupture of the left radial artery, that required emergent surgical treatment.

## CASE DESCRIPTION

A 70-year-old man with ischemic cardiomyopathy in New York Heart Association class III (left ventricular ejection fraction = 35%), previous acute myocardial infarction, body mass index of 26.8 kg/m^2^, and hypertension was admitted in our Hospital for life-threatening arrhythmias and increased troponin I levels. The patient was in permanent atrial fibrillation. Oral anticoagulation with warfarin had been interrupted, and the international normalized ratio (INR) at hospital admission was normal (INR = 1.28). An Allen test was performed, then the left radial artery was cannulated with a 6 French, 25 cm long radial sheath (Radifocus Introducer II; Terumo Corporation, Tokyo, Japan). Heparin (70 IU/kg) and acetylsalicylic acid (ASA) 500 mg were administered per iv bolus. Coronary angiography with an unsuccessful attempt to reopen an occluded circumflex artery was performed without complications. The radial sheath was removed immediately after the procedure and patent hemostasis was achieved through an inflatable TR Band (Terumo Corporation, Tokyo, Japan), which was gradually released over 2 hours while the access site was monitored, and was finally removed 4 hours after its application, having achieved a stable hemostasis. The day after, during the implantation of a cardioverter defibrillator, a periprocedural bleeding occurred that was managed applying a TR Band, kept at low inflation pressure for 12 hours. No clinical signs were evident during the inflation period, such as pale, pain, skin injury, or local swelling, and the radial pulse was present all the time. The patient was discharged 5 days later on the following therapy: enalapril, carvedilol, warfarin, pravastatin, furosemide, and spironolactone.

**FIGURE 1:**
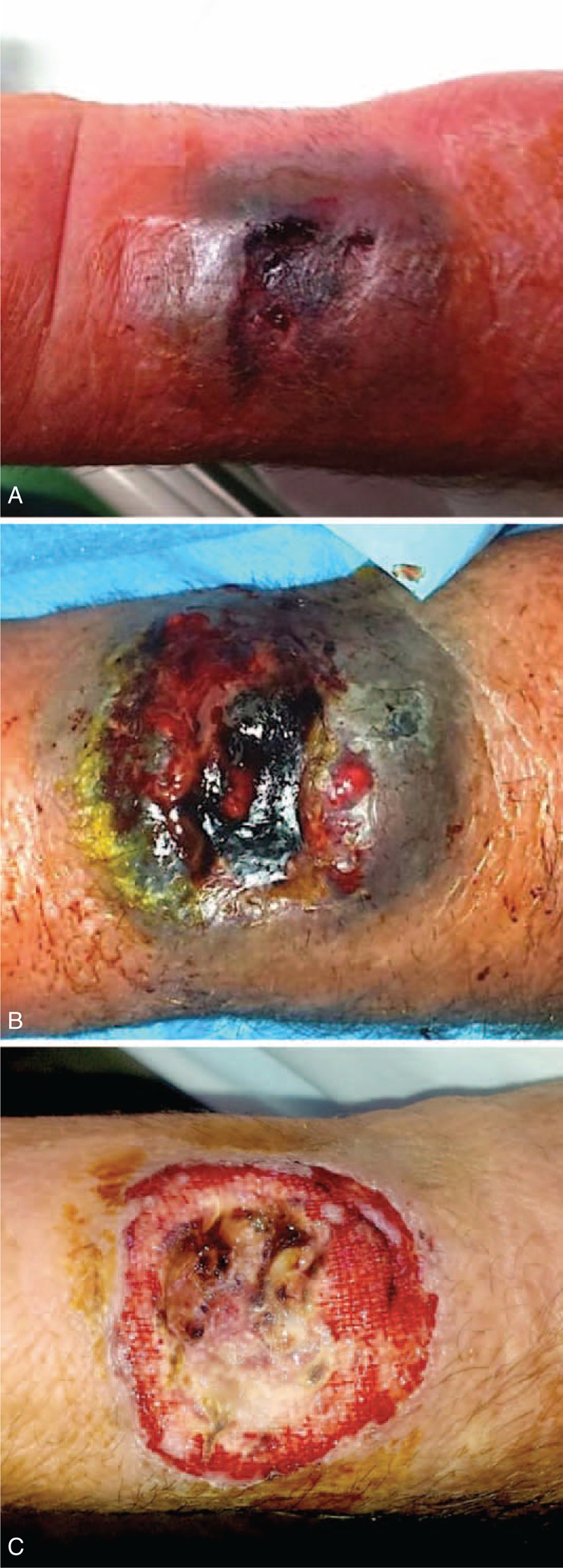
(A) Radial artery puncture site at patient readmission. (B) Aspect of the wound 24 hours after broad-spectrum antibiotic therapy. (C) Appearance of the lesion after wound care and specific antibiotic therapy (3 hours before the radial artery rupture).

**FIGURE 2:**
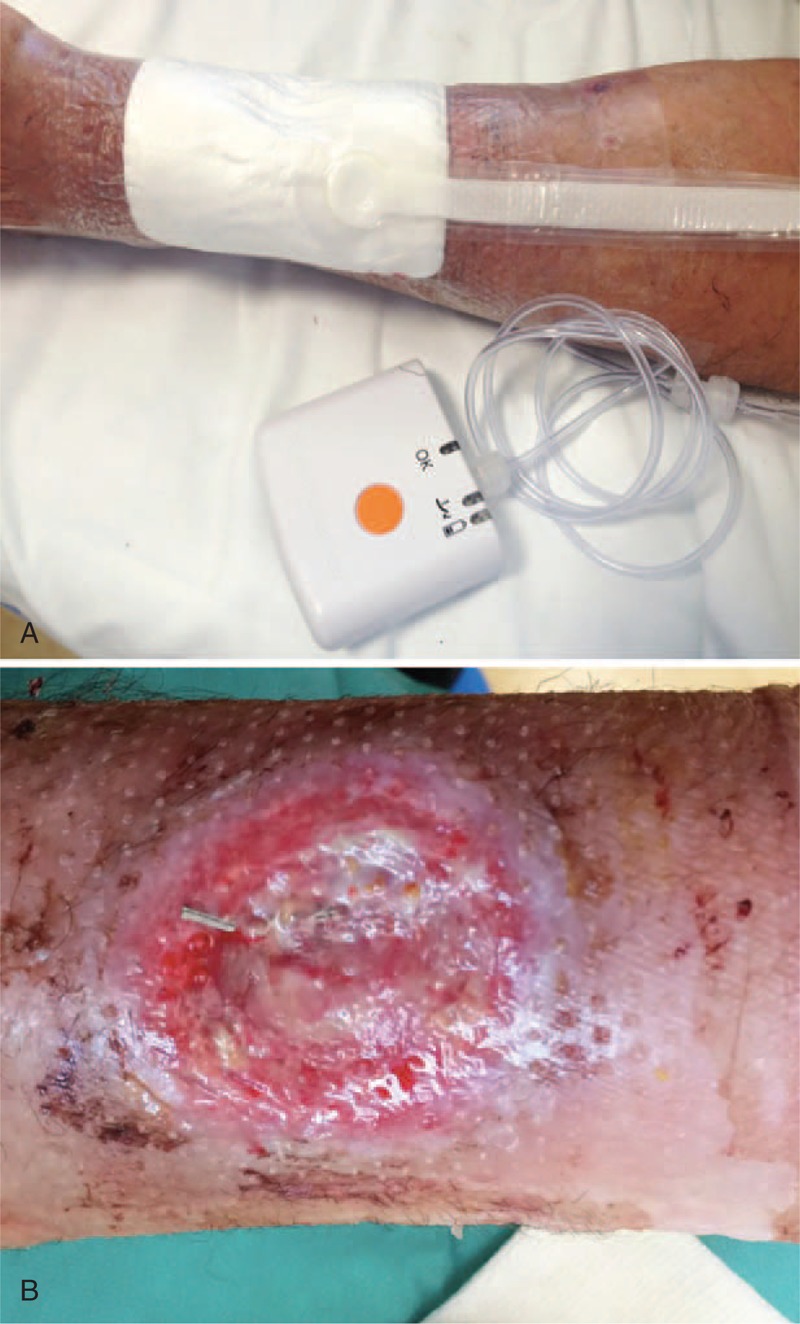
(A) The NPWT device on site. (B) The wound as appeared at the time of PICO removal, after 7 days of NPWT. NPWT = negative pressure wound therapy.

**FIGURE 3:**
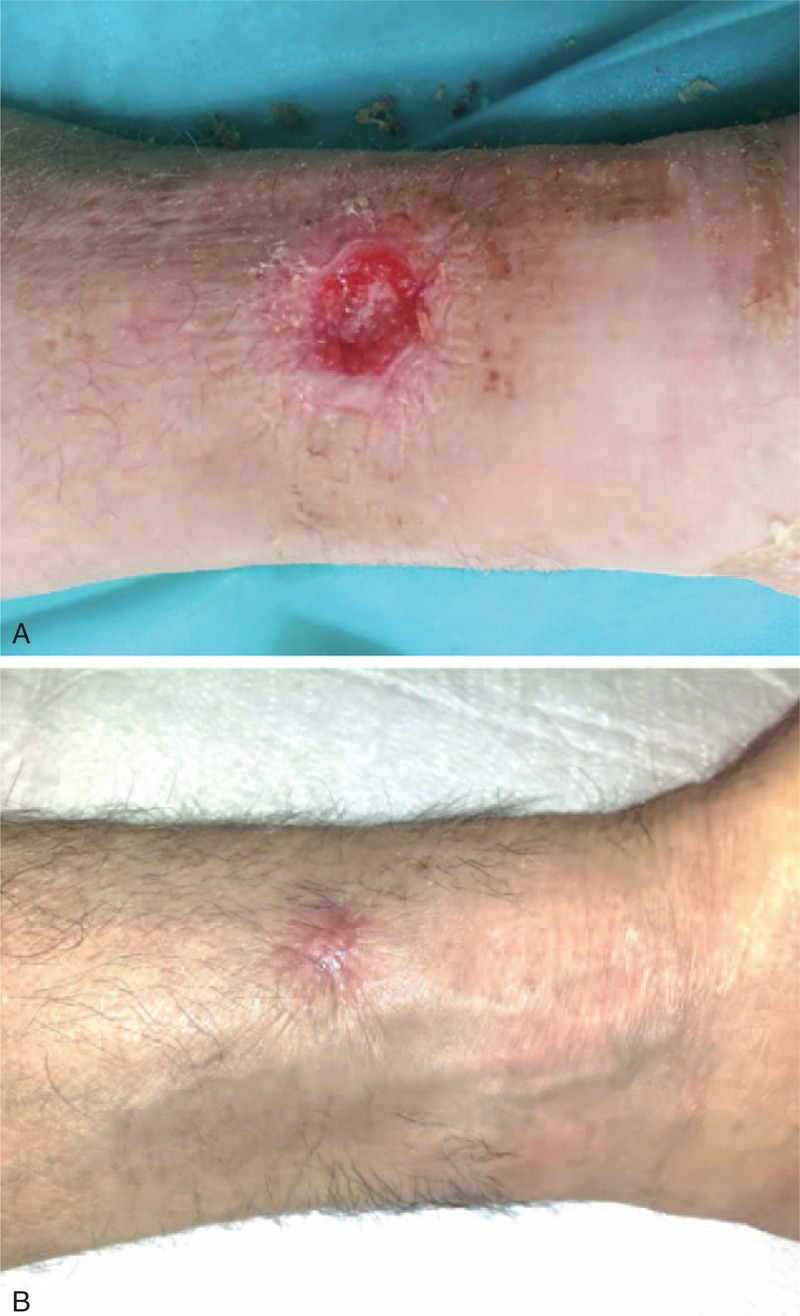
(A) Seven-day follow-up visit. (B) 30-day follow-up visit.

Three days after hospital discharge, the patient referred a pain at his left wrist. The examination of the radial puncture site revealed an exudating ulceration with an underlying hematoma (Figure 1A). The patient was pyretic (38°C) and referred to have fingered and scratched the previous access site. Because the INR was still low (INR = 1.14), the patient was on low molecular weight heparin at this time. Analgesics and an empirical broad-spectrum antibiotic therapy with teicoplanin 200 mg/day and levofloxacin 500 mg/day were started (Figure 1B). A specimen of the exudate was collected for pathogen isolation, revealing an infection by *Staphylococcus aureus*, leading to modification of the antibiotic treatment. Thirty-six hours later, after standard wound care and a specific antibiotic therapy with meropenem 2000 mg three times a day and linezolid 600 mg twice a day, the patient became asymptomatic (Figure 1C). However, a pulsing swelling was observed at the infection site, and duplex sonography documented a patent radial artery without any pseudoaneurysm or intravascular thrombosis. Three days later (15 after TRA), a sudden massive bleeding from the wound occurred. Immediate hemostasis was achieved through manual compression. An emergent surgical exploration of the lesion revealed a gross rupture of the radial artery, with evidence of a long longitudinal laceration, not amenable of repair, which was treated with radial closure. Due to the significant loss of substance, the wound was left to heal by secondary intention with the application of a negative pressure wound therapy (NPWT) device (PICO, Smith & Nephew, London, UK) that was very effective in speeding up the recovery process (Figure 2A). After 7 days of NPWT, a progressive healing of the lesion was observed (Figure 2B), that was even more evident over the following weeks (Figure 3A), with complete restitutio ad integrum at 1 month (Figure 3B).

### Informed Consent

Written informed consent was given by the patient. No approval by an ethical review board was required for this case because the patient was management by usual care.

## DISCUSSION

Major vascular complications at the access site (hematomas, pseudoaneurysms, arteriovenous fistula, arterial dissection, and bleedings) are relatively rare after TRA. The most common local complications are radial artery spasm during catheterization and postprocedural occlusion.^3,4^ Access site infections are even more rare.^5^ We report the first case of gross radial artery rupture associated with local infection 15 days after a transradial endovascular procedure. At inspection, a macroscopic injury of the arterial wall was manifest, with a long longitudinal gross laceration. Fortunately, the patient was at the hospital, and therefore an urgent surgical hemostasis could be promptly performed.

As reported in the case description, we used a left transradial approach (LRA) for the catheterization procedure because the LRA is the default approach for coronary procedures in our institution, due to the excellent safety profile. In fact, we recently demonstrated that the use of the LRA is associated with a significant reduction in fluoroscopy and total procedural time, as well as to a decrease in the amount of contrast medium administration, over the right radial approach.^6^ An additional advantage of the LRA is preservation of the right hand in case of serious complications.

A higher ASA loading dose was administered to the patients at the time of percutaneous coronary intervention. Although this dose was shown to have beneficial effects on thromboxane B2 inhibition and reperfusion indices, it might have exposed the patient to an increased bleeding risk.^7–9^

The major teachings we can learn from this case are as follows: longer or repeated applications of hemostatic devices can increase the risk for local complications, hence particular care should be paid to remove the hemostatic band as soon as possible to avoid tissue necrosis with eventual loss of substance, increasing the risk for arterial rupture; infection of the access site by *S aureus* could facilitate tissue necrosis, especially in the presence of a long-standing hemostatic compression; the use of NPWT can be helpful in favoring and speeding up the healing process in the case of large loss of substance at the infection site; the identification of *S aureus* as the specific pathogen highlights the importance of proper management of access site wound, and the patient should be instructed to manage the site of previous access with care for the first days after discharge.
